# Layered ZnO/MgO Nanohybrids for Photocatalytic Caffeine Degradation

**DOI:** 10.1021/acsomega.5c11898

**Published:** 2026-02-10

**Authors:** Lorena Portela Brazuna, Benjamim Sipauba Gonçalves Rubim, Rebeca Bacani, Josy A. Osajima, Eduardo Rezende Triboni

**Affiliations:** † Universidade Federal de São Paulo, Campus São José dos Campos Unidade Talim, Rua Talim, n° 330, Vila Nair, São José dos Campos, São Paulo Cep 12231-280, Brazil; ‡ Laboratório Interdisciplinar de Materiais Avançados (LIMAV), Universidade Federal Do Piauí, Campus Ministro Petrônio Portela, Teresina, Piauí Cep 64049-550, Brazil; § 119119 Escola de Engenharia de Lorena da Universidade de São Paulo, Departamento de Engenharia Química (DEQUI), Estrada Municipal do Campinho, 100, Lorena, São Paulo Cep 12602-810, Brazil

## Abstract

This study presents the successful development of layered ZnO/MgO nanohybrids via a novel, sustainable one-pot, low-cost, low-temperature route using combined Zn-based (glycerol–urea) and Mg-based (isopropanol–glycerol) hydrogels. This method represents a significant advance in green chemistry by yielding a high-performance hybrid material without the use of noble metal nanoparticles or toxic surfactants. Comprehensive analysis using X-ray diffraction (XRD), electron microscopy (TEM/SEM), and UV–vis spectroscopy confirmed the formation of a ZnO/MgO heterostructure dominated by nanorods. The nanorod morphology offers structural advantages by increasing the specific surface area and minimizing agglomeration, essential for maximizing catalytic efficiency. Furthermore, microstructural analysis revealed that the optimal ZM 3–1 sample possesses high dislocation densities in both crystalline phases, validating the engineered electronic properties and suppressed crystalline defects required for enhanced photocatalytic activity. When evaluated using caffeine degradation as a technical metric, and since it is an emergent pollutant, the ZM 3–1 nanohybrid demonstrated a substantial synergistic effect, achieving 64% degradation in 120 min, which is nearly two times greater than the efficiency of pure ZnO. This superior performance resulted in significantly accelerated reaction kinetics, comparable to those reported for conventional noble metal-based photocatalysts, highlighting the technical merit of this metal-free, structurally optimized material for advanced oxidation processes.

## Graphical abstract

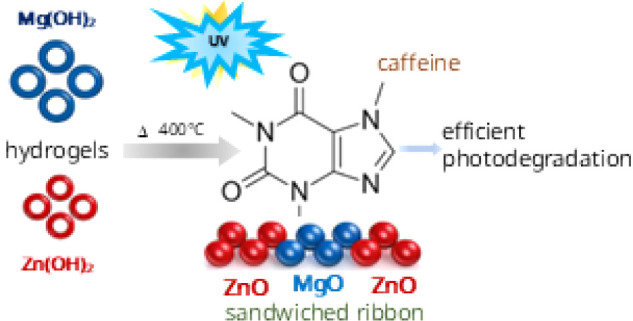

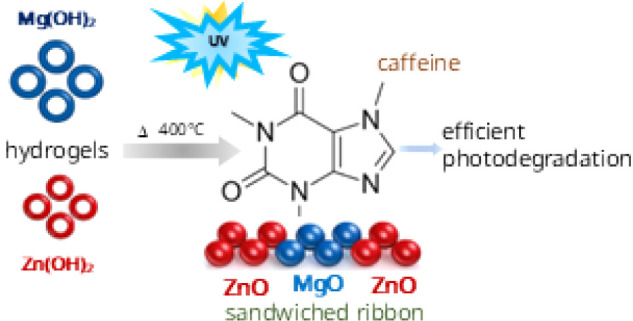

## 1 Introduction

Caffeine is widely used in beverages and pharmaceutical products, and has emerged as a contaminant of concern in water bodies worldwide. Its persistent presence in the aquatic environment is primarily due to human excretion, as approximately 10% to 20% of ingested caffeine is excreted unchanged in urine. Moreover, conventional sewage treatment plants often fail to eliminate it, resulting in its dispersion into rivers, lakes, and even groundwater.^1–3^ The environmental issue linked to caffeine extends beyond its mere detection. As a biologically active substance, it can induce adverse effects in aquatic organisms even at low concentrations.^4–7^ Research indicates that chronic exposure to this compound can lead to metabolic changes in fish, endocrine disruption in amphibians, and oxidative stress in microalgae. In coral reefs, for instance, significant levels of caffeine have already been linked to bleaching and reduced biodiversity.^8–10^ In addition, caffeine can act as an indicator of the presence of other emerging contaminants, such as pharmaceuticals and personal care products, which frequently coexist in urban effluents.^11,12^ The persistence of caffeine in public water supplies can be a of growing potential health effects on humans, although the concentrations typically found in drinking water are low enough to cause direct effects, the presence of caffeine indicates possible contamination of water by untreated sewage, which may serve as a vector for other, more hazardous contaminants, so the efficient removal of caffeine from aquatic systems continues to pose a growing challenge.^11^ Conventional treatment methods, such as activated sludge and filtration, show limited effectiveness, particularly in treatment plants with high organic loads.^9^ More effective alternatives include advanced oxidative processes, such as ozonation and photocatalysis, which can break down the molecule into less harmful byproducts.^8^

In this context, nanomaterials and nanocomposites such as doped metal oxides, supported nanoparticles, and hybrid structures have enhanced degradation of caffeine in wastewater due to the light absorption, increase surface area, and facilitate more effective.nanotechnology can enhance the efficiency of photocatalytic caffeine removal through^11–14^ Caffeine can be eliminated from water via photocatalysis using nanoparticles, particularly when modified with metals or supported on various substrates. For instance, Pham, Chu, and Vu^15^ demonstrated significant caffeine removal efficiency with Mg-doped Zn–Al_2_O_3_ heterostructures. Sathish et al.^14^ investigated the photocatalytic degradation of caffeine using Co–Zn/Al_2_O_3_ nanocomposites, showing its effectiveness in decomposing this pollutant. ZnO and MgO are considered excellent photocatalysts because they combine high photocatalytic efficiency with environmental safety, unlike many other metal oxides that risk leaching toxic byproducts.^16^ Both are wide band gap semiconductors (ZnO = 3.2 eV, MgO = 5.8 eV) capable of generating reactive oxygen species such as hydroxyl radicals and superoxide anions under light irradiation, which effectively degrade organic pollutants into harmless end products.^17^ Their stability, recyclability, and ability to reduce electron–hole recombination, especially when used as ZnO/MgO composites, further enhance their photocatalytic performance.^18,19^ Importantly, they are nontoxic, approved for safe use in biomedical applications, and do not act as pollutants themselves since they remain chemically stable, insoluble under environmental conditions, and do not release harmful ions. This makes them sustainable, green materials for wastewater treatment and pollutant degradation.^20^

Our research group has developed a new, fast, easy, low-cost, low-temperature, one-pot, and reproducible synthesis of ZnO/MgO hybrid nanoparticles (NPs). In this work, ZnO/MgO nanohybrids were investigated for the removal of caffeine via photocatalysis, aiming to create a nanomaterial with suitable photocatalytic properties, as no articles have explored such nanomaterials from hydrogels. The crystallinity and structure of the NPs were verified by a comprehensive XRD analysis combined with SEM and TEM microscopy. UV–vis measurements were studied to determine their optical properties. The photocatalytic activity of the fabricated nanohybrids was investigated by the degradation of caffeine under ultraviolet light. To the best of our knowledge, the use of ZnO/MgO hybrid nanomaterials without metal nanoparticles for caffeine degradation has not been reported previously. This novel and environmentally friendly synthesis exhibits multifunctional characteristics that can be applied to other applications.

## 2 Experimental Section

### 2.1 Chemicals

All reagents are of analytical purity (P.A.) and were obtained from the Synthlab company and used as purchased. The water employed was double-distilled and deionized through a reverse osmosis system. Reagents: zinc nitrate hexahydrate (Zn­(NO_3_)_2_·6H_2_O), magnesium nitrate hexahydrate (Mg­(NO_3_)_2_ 6H_2_O), urea (CH_4_N_2_O), isopropanol (C_3_H_8_O), glycerol (C_3_H_8_O_3_), sodium hydroxide (NaOH), and double-distilled water (H_2_O).

### 2.2 Synthesis of Zn-Based Hydrogel

For the synthesis of the Zn-based gel, the GU route described in Brazuna et al.^21^ was used. The glycerol:urea in 3:1 mol was stirred at 70 °C, then set to cool at room temperature. Then 0.05 mol Zn­(NO_3_)_2_·6H_2_O was added to the solution, stirred for 15 min, and 0.05 mol of NaOH was added, under stirring and at room temperature for another 15 min. The gel was washed with 600 mL of deionized water and centrifuged four times.^22^

### 2.3 Synthesis of Mg-Based Hydrogel

For the synthesis of the Mg-based gel, the GU-based route described in Brazuna et al.^21^ was used, with urea replaced by isopropanol. The glycerol:isopropanol 3:1 mol was stirred at room temperature, then 0.05 mol Mg­(NO_3_)_2_·6H_2_O and 0.01 mol NaOH were added, stirred for another 15 min. The gel was washed with 600 mL of deionized water and centrifuged four times.^23^

### 2.4 Synthesis of ZnO/MgO Nanohybrids

The as-prepared hydrogels were combined in three mass ratios (3:1, 5:1, and 10:1 Zn:Mg) and homogenized. The pure and hybrid materials were then calcined in a muffle furnace at 400 °C for 3 h to obtain the corresponding pure ZnO, MgO, and ZnO/MgO nanohybrids. The final products were designated as **ZnO** (pure nanozinc oxide), **ZM 3–1**: ZnO/MgO (3:1 w/w), **ZM 5–1**: ZnO/MgO (5:1 w/w), **ZM 10–1**: ZnO/MgO (10:1 w/w), and **MgO** (pure nano magnesium oxide). The final powder has an average of 0.6 g per sample. The **ZM 20–1**: ZnO/MgO (20:1 w/w) nanohybrid was also synthesized for morphological investigation (Figure S5).

### 2.5 Characterization of the ZnO/MgO Nanohybrids

Electron microscopy images were used to investigate the morphology and dimensions of the nanoparticles. Scanning Electron Microscopy (SEM) images were acquired using a JEOL JSM7401F field-emission electron microscope operating at 5.0 kV. A secondary electron detector (SEI) with a working distance of 3.0 mm and a resolution of 1.5 nm was employed. For nanoscale analyses, a high-resolution transmission electron microscope (HRTEM) JEM-2100F, JEOL, with a point resolution of 0.23 nm and an accelerating voltage of 1.5 kV was utilized. Energy-dispersive X-ray spectroscopy (EDS) was utilized to assess nanomaterial atomic heterogeneities and perform atomic analysis, coupled with a JEM-2100 JEOL. TEM, HRTEM, and, when possible, SEM images were analyzed using ImageJ software to determine the average size of the nanoparticles in the samples.

X-ray diffraction (XRD) was conducted on dry nanoparticles in powder form using a PANalytical Empyrean diffractometer equipped with a PIXEL3D detector. The radiation was produced by a copper tube (λ = 1.5418 Å) with a nickel filter. The operating conditions included a voltage of 40 kV and a current of 30 mA, with angular scanning (2θ) ranging from 10° to 80° in 0.02° increments and an acquisition time of 5 s per step. The identification of the crystalline phases was carried out by comparison with the ICDD and ICSD databases, utilizing the Malvern PANalytical HighScore Plus software.^24^ Structural analysis was conducted by crystalline phase indexing by databases, the Rietveld method, and the Williamson-Hall plot. The Rietveld method was employed to calculate the diffraction pattern and obtain structural parameters, including cell lattice parameters and cell volume. A pseudo-Voigt function was used for whole profile analysis, incorporating scale fraction, temperature factors, and a sixth-degree polynomial background as fitted parameters. This method was also applied to assess the potential for Mg-doping through occupancy factor refinement. The occupancy factors indicate the fraction of a specific crystallographic site occupied by an atom, in this case, Mg on the Zn site, to account for deviations from stoichiometry, such as partial occupancy due to disorder or vacancies. Williamson-Hall was utilized to determine the average crystallite size and microstrain for each sample. Strain analysis is crucial for nanocrystalline systems, as the Scherrer formula has size limitations for crystallites smaller than 100 nm due to the broadening of the diffraction peaks. Williamson–Smallman dislocation densities were also calculated using whole-profile analysis.^25–29^

UV–vis absorption measurements were conducted using an FS5 spectrometer (Edinburgh Instruments). For sample preparation, 1 mg of nanoparticles was dispersed in 10 mL of double-distilled water through sonication for 30 min. Aliquots of 3 mL were transferred to quartz cuvettes with an optical path of 10.0 mm for spectral recording. The band gap energies (*E*_g_) were calculated from diffuse reflectance data obtained on a Shimadzu UV-3600 spectrophotometer equipped with a reflectance measurement accessory. The analyses spanned the spectral range from 220 to 600 nm, and *E*_g_ values were derived by applying the Tauc plot.^19,30–37^

### 2.6 Photocatalytic Degradation of Caffeine

The study evaluated the photodegradation of caffeine (10 mg L^–1^) using ZnO/MgO hybrids (0.5 g L^–1^) as photocatalysts. The tests were conducted in a 100 mL borosilicate glass reactor coupled to an irradiation system equipped with a mercury vapor lamp (125 W, without bulb), which was maintained at 25 °C by a thermostatic bath. The lamp presented a radiation intensity of 6.0 ± 0.2 μW cm^–2^, measured using a radiometer (Hanna), with a main emission peak in the range of 350–450 nm (Figure S1).^38–41^ Before irradiation, the solutions were stirred in the dark for 30 min to establish the adsorption–desorption equilibrium between the catalyst and the contaminant (Figure S2).^42–44^ The maximum absorption wavelength of caffeine was determined by UV–vis spectrophotometry at a wavelength of 273 nm. A calibration curve was subsequently constructed at this wavelength (Figure S3). The process was monitored by periodically collecting 0.9 mL aliquots at 0, 5, 10, 15, 30, 45, 60, 90, and 120 min.^38,45–51^ The degradation efficiency was calculated according to eq 1:^52,53^

1 $$\mathrm{Removal efficiency}\left(\%\right)=\left(\frac{C_{0}-C}{C_{0}}\right)\times 100$$

The *C*_0_ represents the initial concentration and *C* the concentration at time *t* of analysis. To evaluate the process kinetics, the pseudo-first-order model expressed by was applied:^54^

2 $$\ln\left(\frac{C_{0}}{C}\right)=kt$$

where *k* is the apparent rate constant (min^–1^), and *t* is the reaction time. Additionally, the half-life (*t*_1/2_) corresponding to the period required to reduce the initial concentration by 50% was determined. To evaluate the isolated contribution of radiation to caffeine degradation, a blank experiment (photolysis) was performed under the same conditions, in which only the caffeine solution was exposed to UV light without the presence of the photocatalyst.^38,41,42,53^

## 3 Results and Discussion

### 3.1 Morphological, Structural, and Optical Characterization

The morphology and average size of the ZnO, MgO, and ZnO/MgO nanohybrids were investigated using SEM in Figures 1 and 2 and HRTEM in Figures 3 and 4. In Figures 1(a) and 3(a), the results show the formation of spherical ZnO nanoparticles with an average diameter of 21 nm. In Figure 1(b) and Figure 3(b), MgO nanosheets, with an average length of 13.5 nm, are typical morphology results found in the literature.^21,23,54,55^ In Figures 2 and 4, the addition of MgO to the system resulted in an increase in rod-shaped nanoparticles as the amount of Zn increased, although the system exhibits some nanoheterogeneity. For ZM 3–1 in Figure 2(a) and Figure 4(a), the average rod size was 0.59 μm in length and 0.57 μm in diameter, accompanied by some 16.6 nm spheres. For ZM 5–1 in Figure 2(b) and Figure 4(b), the average rod size was 1 μm in length and 0.57 μm in diameter, along with some 16.6 nm spheres. For ZM 10–1 in Figure 2(c) and Figure 4(c), the average rod size was 0.335 μm in length and 0.051 μm in diameter, with a rare amount of 27 nm spheres.

**Figure 1:**
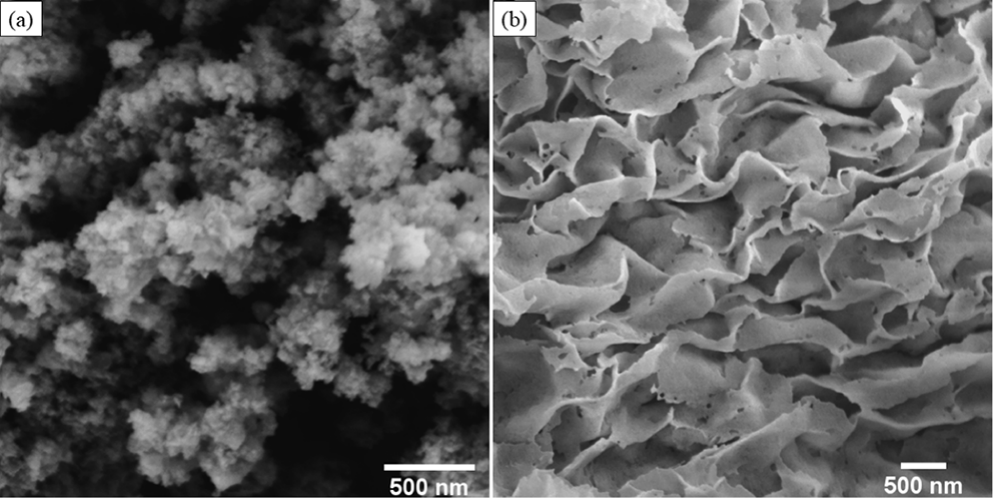
SEM images of the standard nanomaterials: (a) ZnO and (b) MgO.

**Figure 2:**
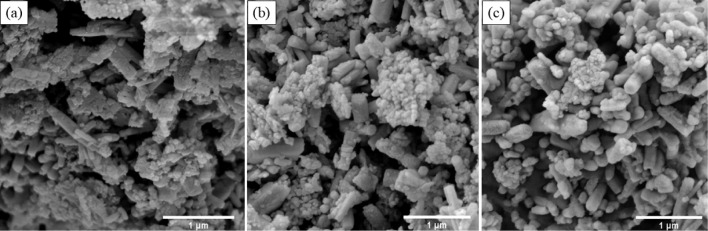
SEM images of the nanohybrids ZnO/MgO: (a) ZM 3–1; (b) ZM 5–1; and (c) ZM 10–1.

**Figure 3:**
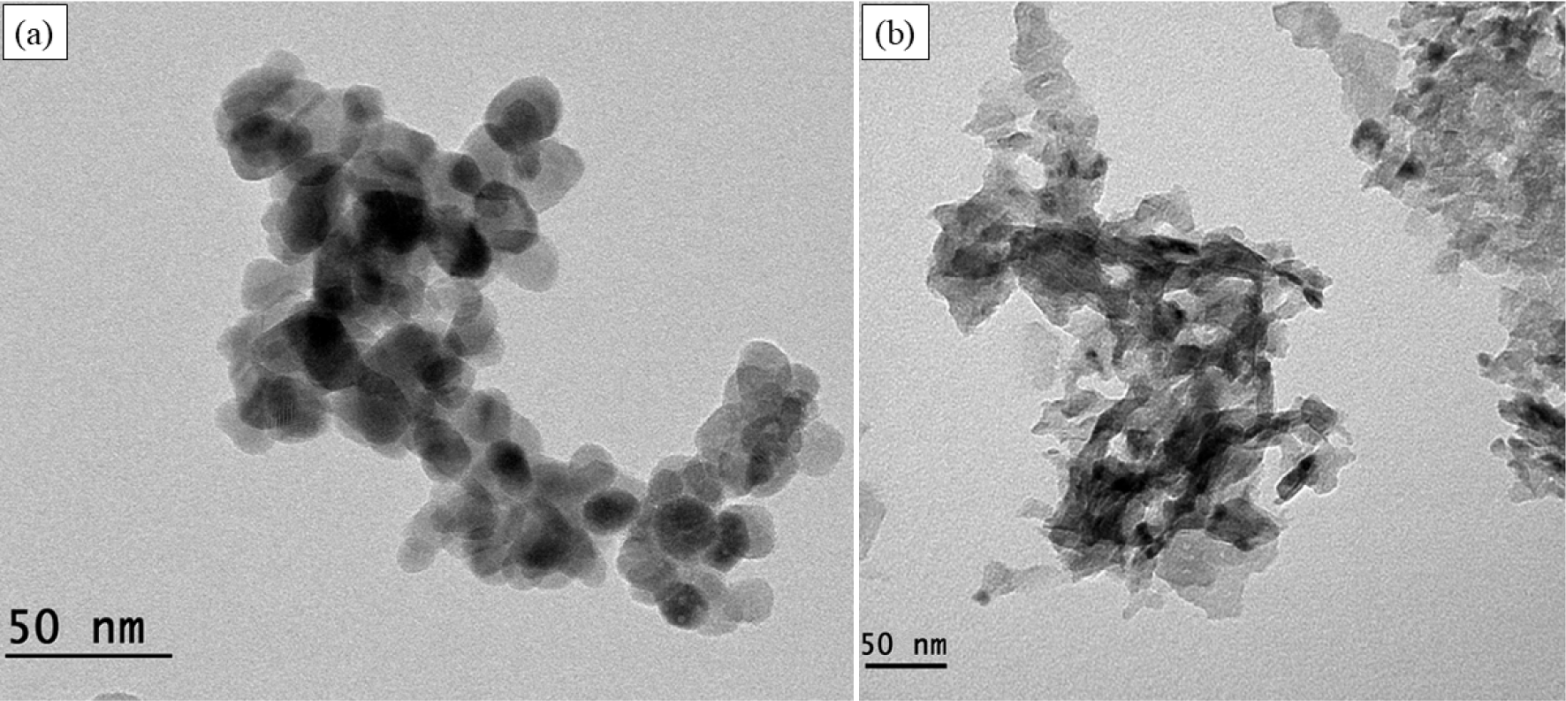
HRTEM images: (a) ZnO and (b) MgO.

**Figure 4:**
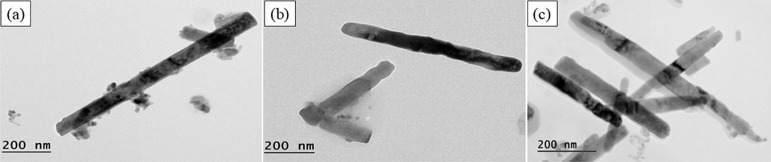
HRTEM images of ZnO/MgO: (a) ZM 3–1, (b) ZM 5–1, and (c) ZM 10–1.

The morphology related to the chemical composition of the ZnO/MgO samples was investigated using TEM and EDS analysis, as shown in the images of Figures 5, 6, and 7. In ZM 3–1 Figure 5(a), the TEM results showed nanorods with the presence of spherical NPs, and in Figure 5(b), the EDS results indicated the heterogeneous presence of ZnO and MgO (while EDS 1 showed MgO presence, in the other section, EDS 2, there is no presence of MgO). In ZM 5–1 Figure 6(a), there are also nanorods and spherical NPs. In (b), the EDS results revealed phase separation between the nanorods and spheres, where the larger rods are predominantly composed of ZnO, and the smaller NPs contain MgO. Moreover, in ZM 10–1 Figure 7(a), the TEM images show the prevalence of nanorods with some spherical NPs. In (b), the EDS results also indicate the presence of Zn and Mg. These results suggest the formation of a heterogeneous composition of ZnO and MgO, with an increase in the presence of nanorods as the amount of Zn in the samples increases.^56^ Another view of other ZM 20–1 materials that we also synthesized shows the intensity difference between both ZnO (darker) and MgO (lighter). There is the formation of a hybrid ZnO/MgO heterostructure. The bandgap change is also small and characteristic of ZnO-based heterostructures (Figure S5).^57,58^

**Figure 5:**
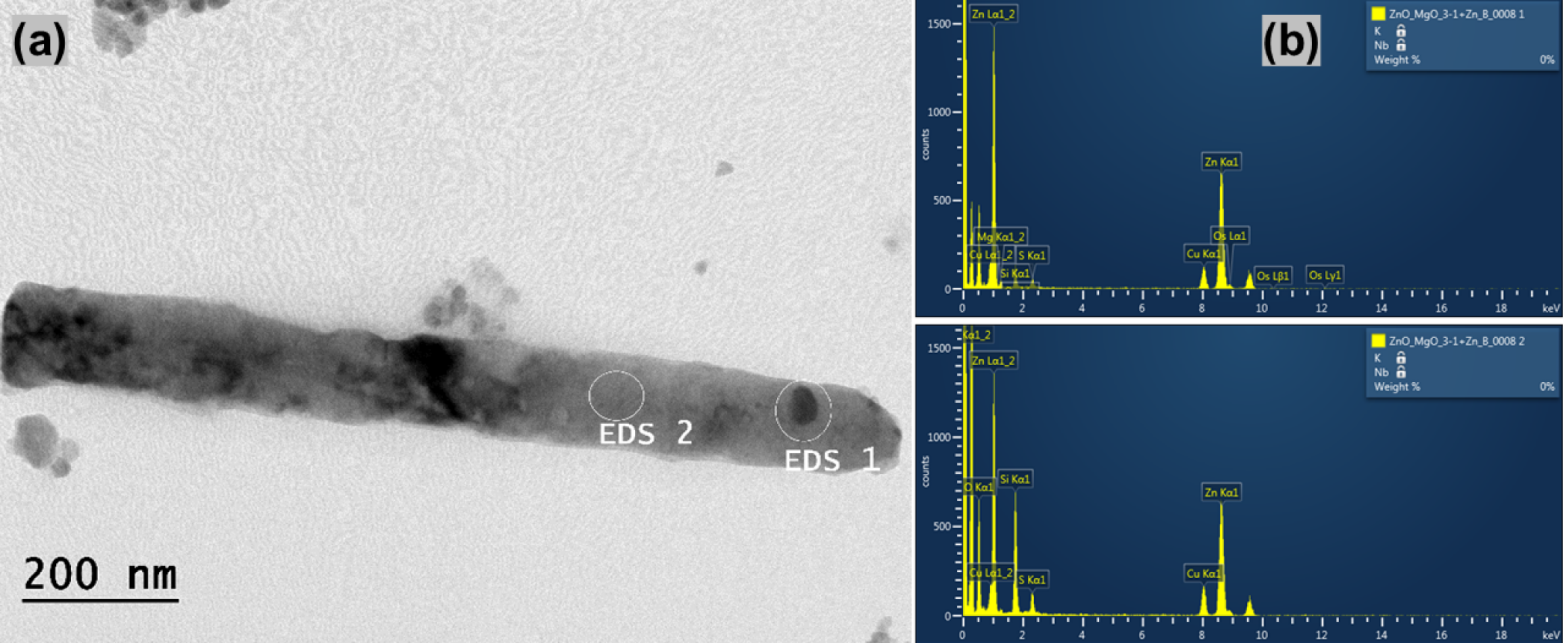
HRTEM/EDS images of ZnO/MgO. ZM 3–1: (a) HRTEM and (b) atomic composition via EDS (enhanced images in the Supporting Information (Figure S4a)).

**Figure 6:**
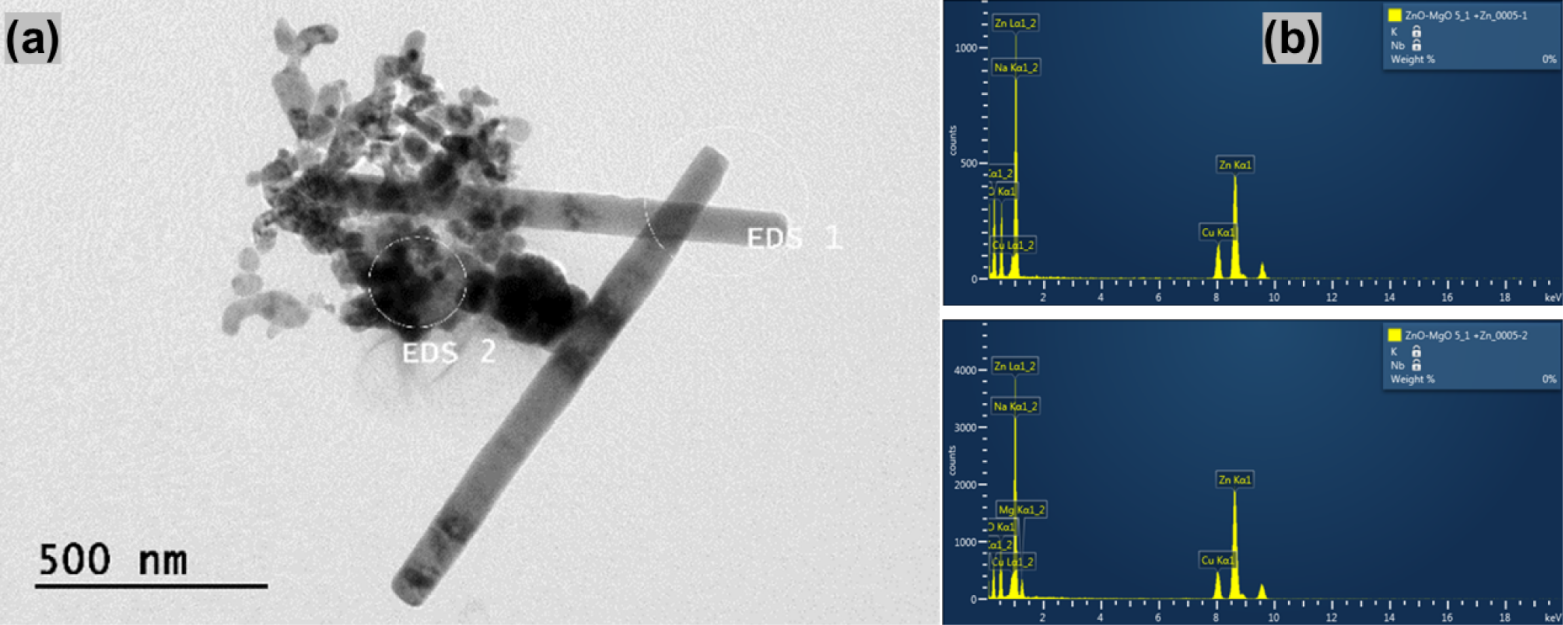
HRTEM/EDS images of ZnO/MgO. ZM 5–1: (a) HRTEM and (b) atomic composition via EDS (enhanced images in the Supporting Information (Figure S4b)).

**Figure 7:**
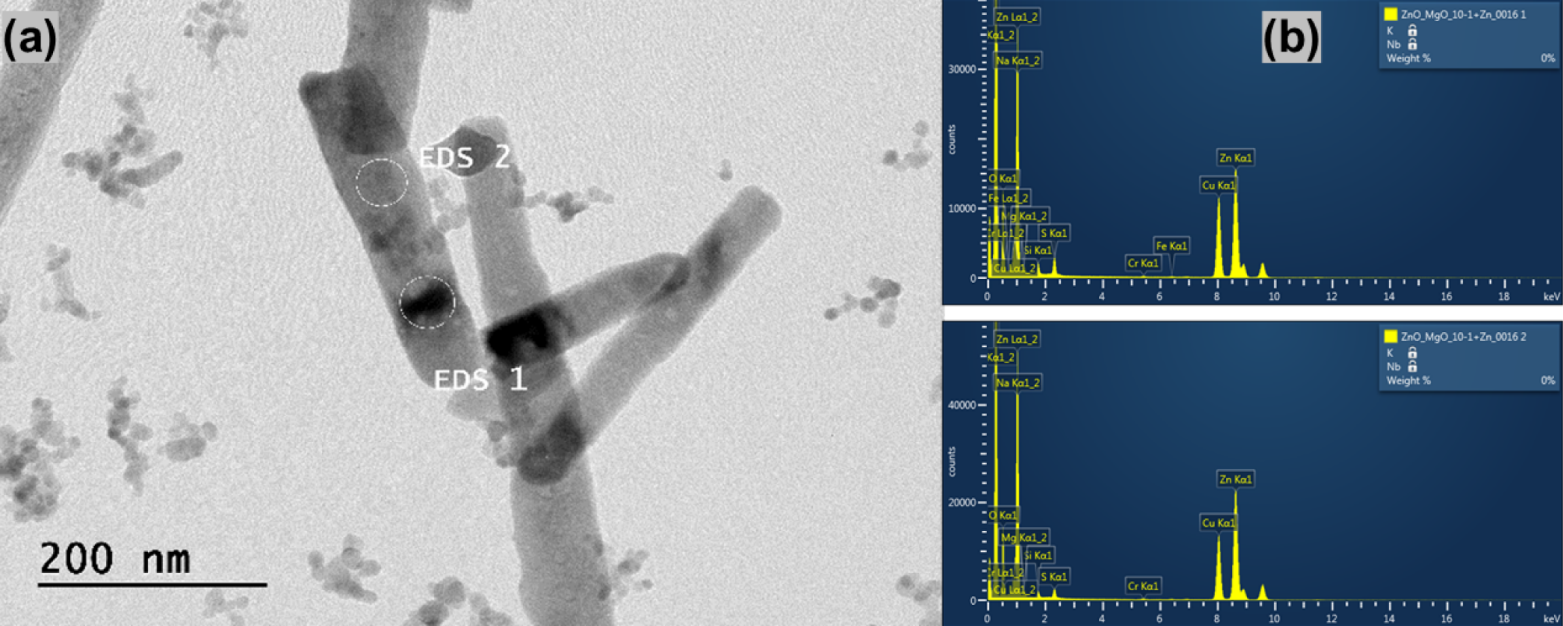
HRTEM/EDS images of ZnO/MgO. ZM 10–1: (a) HRTEM and (b) atomic composition via EDS (enhanced images in the Supporting Information (Figure S4c)).

This distinctive morphological configuration, combined with the electronic synergy between the component oxides, can simultaneously optimize the adsorption of contaminants and the efficiency of generating reactive oxygen species (ROS) during irradiation due to the greater availability of accessible active sites.^59^ Additionally, the one-dimensional architecture of these nanomaterials presents a comparative advantage over conventional spherical morphologies, not only due to the larger specific surface area^60^ but also by minimizing agglomeration phenomena, thus preserving the active sites throughout successive photocatalytic cycles.^61^ Unlike conventional catalysts, such as TiO_2_, in which increasing the surface area often requires doping with metals, the MgO/ZnO system achieves this property intrinsically through its nanostructured architecture.^62,63^

The XRD patterns are shown in Figure 8. All materials showed high crystallinity, and the ZnO was indexed as the hexagonal wurtzite crystal phase (COD number 96–901–1663) and MgO as the cubic periclase phase (ICDD number 96–900–6458).^64,100^ The initial Rietveld model analysis was performed as a multiphase model, with ZnO and MgO, using an occupancy factor for Mg to substitute for the Zn crystallographic site. However, with HRTEM and EDS spectra, evidence of two phases was observed, and the occupancy refinement strategy was abandoned, since layered nanohybrid materials are heterostructured materials composed of a two-dimensional inorganic host and intercalated with inorganic/organic different materials,^65–67^ in this case, resulting in hybrids of ZnO/MgO rods with some spherical particles. Microstructural analysis and the Rietveld results are shown in Table 1. All refinements yielded small *X* ^2^ values, indicating good agreement between the phase model and experimental results.

**Figure 8:**
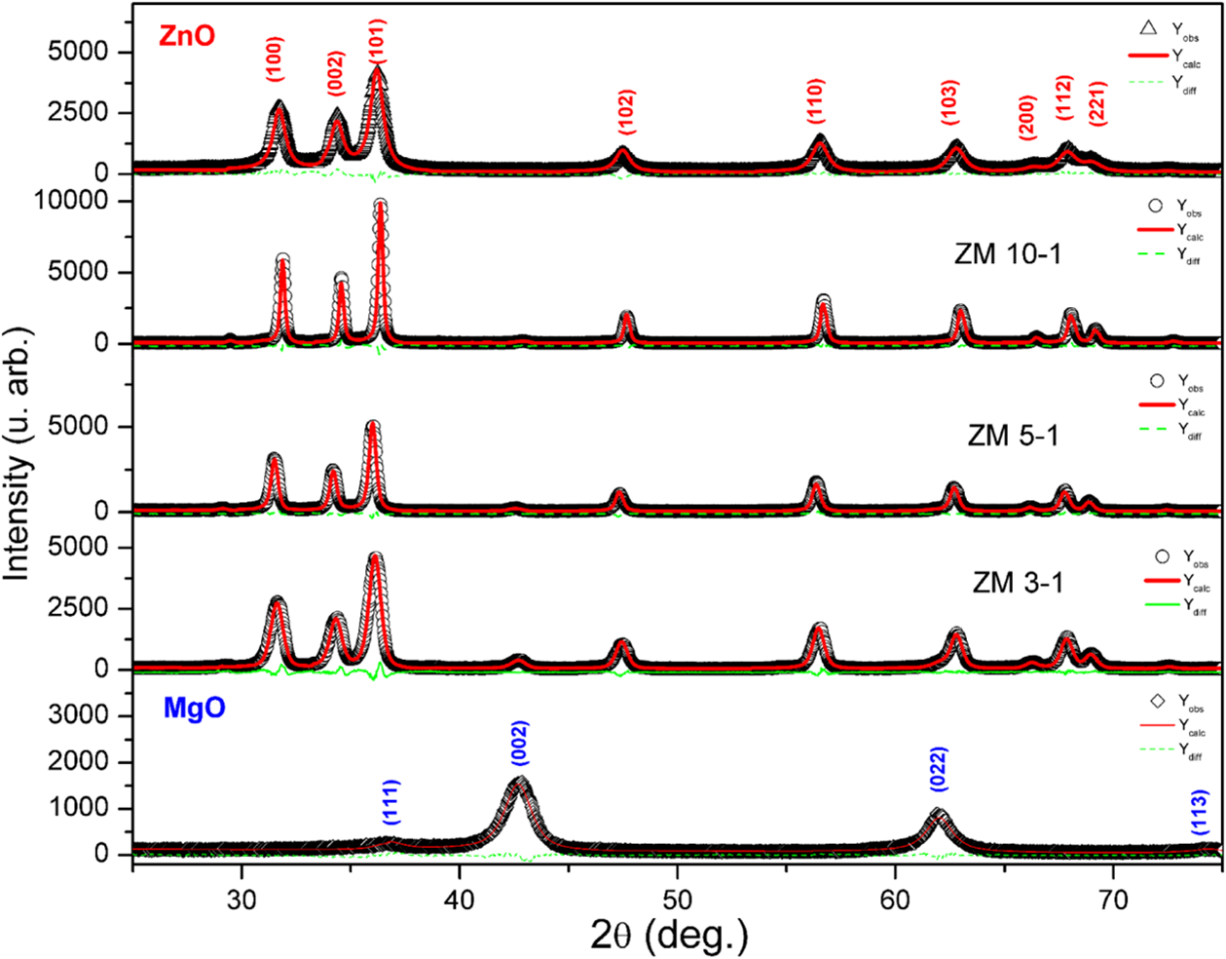
XRD diffraction patterns for all samples. Symbols represent the experimental data points (*Y*_obs_), while the solid lines correspond to the Rietveld refinement calculated profile (*Y*_calc_). The difference plot (*Y*_diff_ = *Y*_obs_ – *Y*_calc_) is shown as dashed lines beneath each pattern.

**Table 1:** Rietveld Method, Williamson-Hall (Crystallite Size and Microstrain), and Williamson–Smallman Results (ρ)^a^

Zn/Mg	phase fractions (%)	*a*,*b*,*c* (Å)	*V* (Å^3^)	ρ (nm^–2^)	Crystallite size (nm)	Microstrain (%)	*X* ^2^
ZM 10–1	MgO 5.4%	*a* = *b* = *c* = 4.209(3)	74.58(7)	0.0059	13(5)	0.813(6)	1.8
	ZnO 94.6%	*a* = *b* = 3.2510(17)	47.65(3)	0.0008	35(5)	0.087(12)	
		*c* = 5.206(3)					
ZM 5–1	MgO 16.0%	*a* = *b* = *c* = 4.2200(8)	75.15(6)	0.0017	24(4)	1.07(3)	1.3
	ZnO 82.2%	*a* = *b* = 3.248(13)	47.48(5)	0.0015	26(3)	0.000015(9)	
		*c* = 5.1979(3)					
ZM 3–1	MgO 26.9%	*a* = *b* = *c* = 4.2167(8)	75.03(4)	0.0051	14(3)	0.0020(3)	1.6
	ZnO 73.1%	*a* = *b* = 3.2497(2)	47.55(5)	0.0083	11(3)	0.0050(10)	
		*c* = 5.1996(4)					
pure MgO	MgO 100%	*a* = *b* = *c* = 4.2269(8)	75.52(5)	0.0278	6.0(8)	0.54(3)	1.2
pure ZnO	ZnO 100%	*a* = *b* = 3.2562(3)	47.8(3)	0.0028	19(3)	0.186(4)	1.6
		*c* = 5.21223(5)					

a Phase fraction (%), lattice parameters (*a*, *b*, *c*), cell volume (*V*). The *X* ^2^ is the final convergence criterion for the Rietveld method. Numbers between parentheses denote the uncertainty, i.e., 0.7(3) means 0.7 ± 0.3.

The bulk lattice cell volume for ZnO is 49.719 Å^3^, and for MgO, it is 74.618 Å^3^. The hybrid nanomaterials synthesized in this work exhibited a smaller volume for the ZnO phase, whereas the MgO phase showed a higher cell volume. In the formation of nanomaterials, whether nanospheres or nanorods, the presence of stress, vacancies, or defects in the bonds between crystalline planes is familiar, which can affect lattice parameters. It is reported that nano-ZnO presents smaller cell parameters as a nanostructure due to surface defects during ZnO formation,^21^ while the cubic structure of MgO is more subject to plastic deformation as magnesium hydroxide oxidizes to MgO, forming flake-like nanostructures,^23^ Figure 1(b).

The material with the smallest amount of ZnO phase presented smaller average crystallite sizes, while for the sample with the most significant amount of ZnO, the crystallite size for the MgO phase is much smaller than for the ZnO phase, showing a greater separation of the phases, which can be observed in the SEM/TEM images (Figures 5, 6, and 7). The microstrain was taken from the Williamson-Hall plot for both phases separately. For the MgO phase, there are no systematic changes; the higher value for the stress in ZM 5–1 could be attributed to the formation of both spheres and rods (Figure 6). For the ZnO, the stress is almost constant, due to the majority formation of nanorods and relaxation of the growth in the *c*-direction, usual of ZnO-based materials.^21,68^ To understand how defects may be associated with the formation of these nanohybrid ZnO/MgO materials, the Williamson’s and Smallman’s formula was analyzed for the dislocation densities, calculated via the relation ρ = *n*/*D* ^2^, where *D* is the average crystallite size, taken from the whole pattern via the Williamson-Hall plot. Taking *n* = 1 gives the minimum dislocation lines per unit volume of the crystal.^69,70^ For nanoparticles and nanosystems, *D* can show lattice imperfections and the presence of lattice defects. While the WH strain showed almost no change for the ZnO phase, the dislocation densities are higher for higher MgO content, which could indicate a higher amount of oxygen vacancies or Mg and Zn interstitial defects. For the 1:3 nanohybrid, both ZnO and MgO phases presented high dislocation densities, which could be tied to higher photocatalytic activity due to defects and vacancies.^44,71^ The microstrain, in this case, could also be related to the nucleation of cubic MgO embedded in zinc oxide.^25–27,71^

Optical characteristics were evaluated through the behavior of the materials in UV–vis spectroscopy. Figure 9 presents UV–vis spectra. UV–vis spectroscopy is an effective technique for predicting the size, shape, and stability of nanoparticles in aqueous suspensions.^72–74^ Nanosized ZnO and MgO synthesized in this work exhibited absorption peaks at approximately 373 and 278 nm, respectively, which align with data reported in the literature.^75,76^ For Zn/Mg nanohybrids, similar findings were observed, with absorption peaks for ZnO ranging from 373 to 374 nm and for MgO from 278 to 279 nm, indicating a heterogeneous structure.^77^ The Tauc Plot method was utilized to estimate and visualize the approximation of the energy transition from the valence band to the conduction band. This model is based on defect-free semiconductors exhibiting a single transition, often a direct transition like ZnO.^19,32–37,78^ The energy band gap values of the ZnO/MgO estimated from the Tauc plot are pure MgO (5.758 eV), pure ZnO (3.286 eV), ZM 10–1 (3.300 eV), ZM 5–1 (3.306 eV), and ZM 3–1 (3.299 eV).

**Figure 9:**
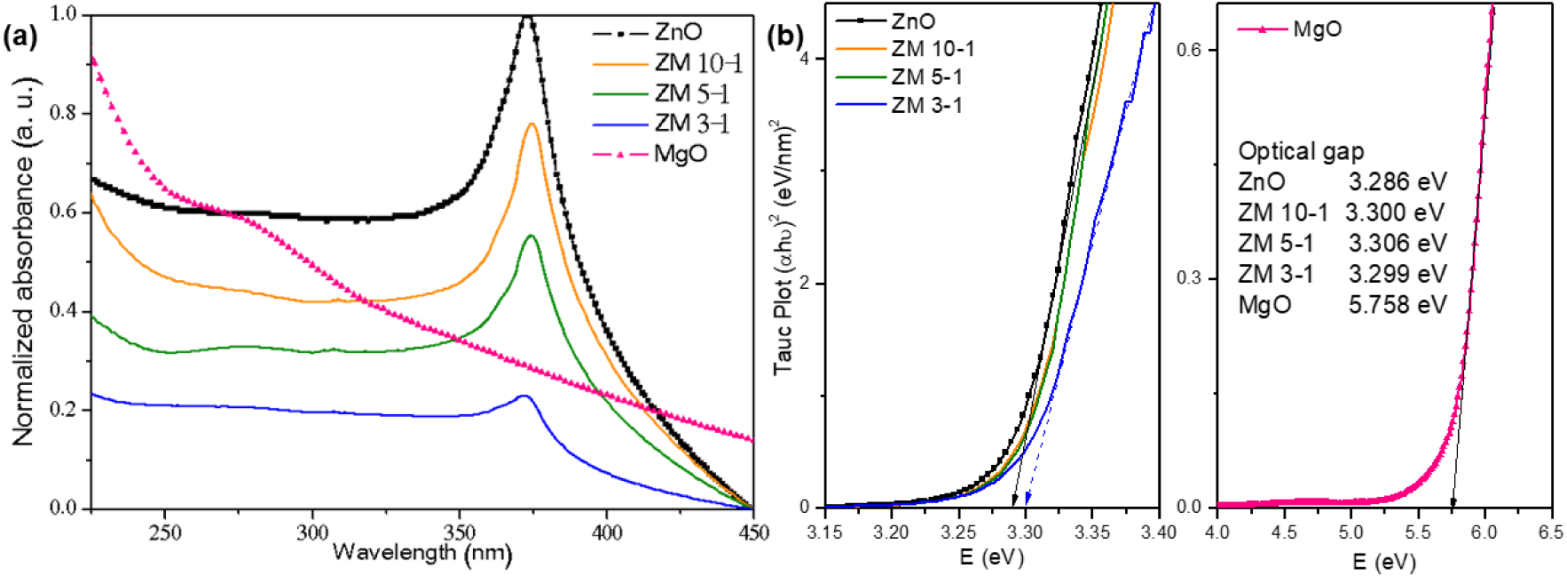
(a) UV–vis spectra of ZnO, ZM 10–1, ZM 5–1, ZM 3–1, and MgO. (b) Energy of band gap: ZnO, ZM 10–1, ZM 5–1, ZM 3–1, and MgO.

Those values for ZM 10–1 and ZM 5–1 were close to pure nano-ZnO but exhibited a decrease for ZM 3–1, which could indicate a change in its local absorption under UV light. This modification of the local spectra can suggest that electrons can be promoted from the valence band to the conduction band with a lower energy supply. Nazim et al.^79^ found similar results with Mg doping in ZnO, also decreasing its band gap value from 3.34 to 3.21 eV. In the synthesis of nanosystems, the presence of stress in nanorods promotes changes in local optical properties, morphology, and band gap energy (direct). In this case, MgO may contribute to the average optical changes on the hybrid nanomaterials, which could be significant for photocatalytic applications. The presence of MgO induces alterations in the optical behavior of the hybrid nanomaterials (Figure 9(b)).

### 3.2 Caffeine Photodegradation

The ZnO/MgO nanohybrids were utilized to assess the degradation of caffeine as a contaminant. Figure 10 illustrates the graphs of caffeine concentrations over time, corresponding to the intervals when aliquots were taken for analysis. The period from −30 to 0 min represents the duration during which the MgO/ZnO nanohybrids were in contact with the contaminant in the reactor without exposure to light. The removal or decontamination rate during this interval shows a decline in the graph, indicating the adsorption of caffeine molecules onto the surface of the photocatalysts. From time 0 to 120 min, ultraviolet light radiation is introduced, providing sufficient energy to excite electrons from the valence band to the conduction band, resulting in the formation of electron–hole pairs. These electron–hole pairs interact with both water and oxygen molecules, producing superoxide and hydroxyl radicals, which are capable of degrading caffeine molecules through oxidation–reduction reactions.^80,81^ Following this activation, the caffeine degradation process commences, with the photocatalysts displaying varying performance; ZM 3–1 shows a more significant reduction in concentration from the initial to the final stage, while others exhibit a more gradual decline. These results suggest that the caffeine concentration decreases within the system due to the degradation effect of the photocatalysts, with the lowest final caffeine concentration observed in the ZM 3–1 sample.

**Figure 10:**
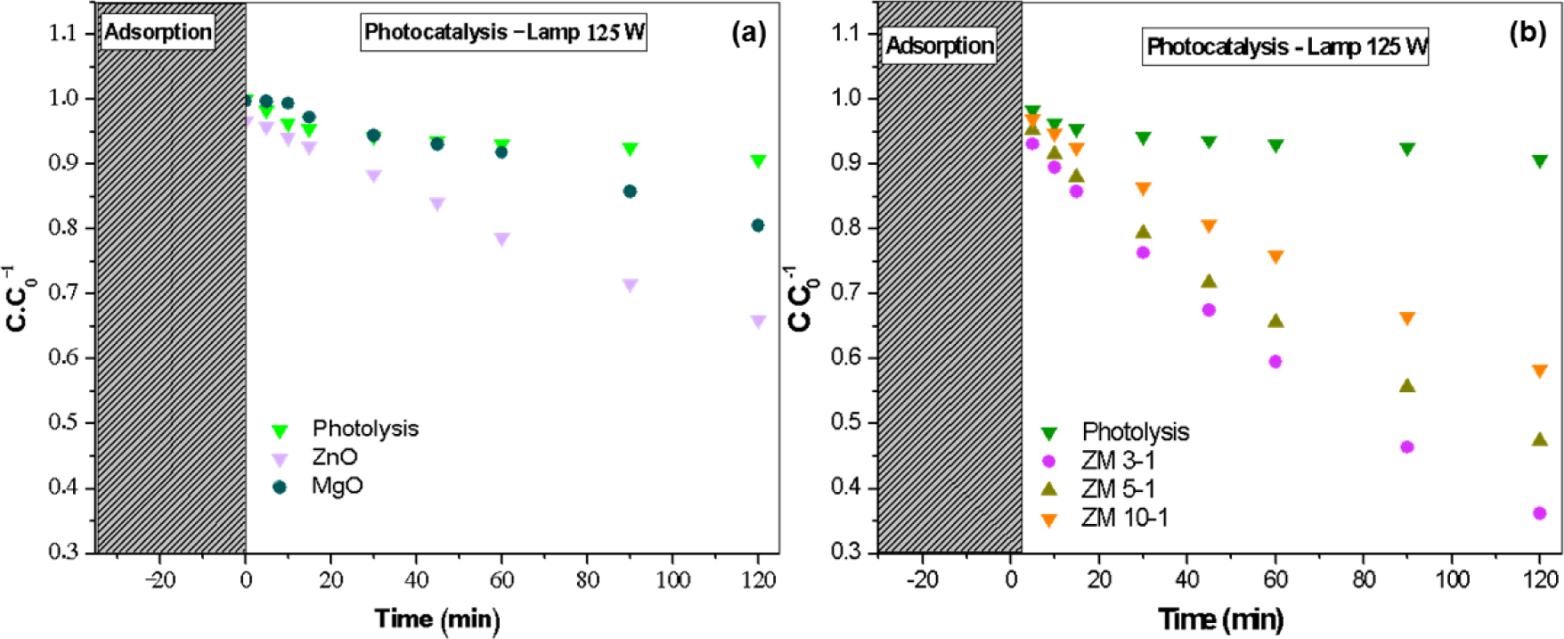
Caffeine concentrations over time, corresponding to the intervals when aliquots were taken for analysis for all materials: (a) Photolysis, pure nano-ZnO and nano-MgO, and (b) Photolysis, ZnO/MgO nanohybrids ZM 3–1, ZM 5–1, and ZM 10–1.

The reaction kinetics behavior was obtained for all photocatalysts, as shown in Figure 11, which reveals a correlation coefficient *R* ^2^ of ∼1 for all samples. This indicates a strong and consistent linear pseudo-first-order relationship, consistent with that found in the literature for nanoparticles formed in heterostructured systems. Nanomaterials formed by heterostructured systems generally exhibit pseudo-first-order kinetics in photocatalytic degradation reactions.^82^ Multiple studies on various heterostructured nanocomposites such as Ag/GO/ZnO, MgO/ZnO/Graphene, ZnO/ZnFe_2_O_4_, and α-MoO_3_/ZnO report the degradation of organic pollutants such as dyes and pharmaceuticals follows a pseudo-first-order kinetic model, as indicated by the linear relationship between the logarithm of the pollutant concentration and the reaction time during photocatalysis under UV or visible light irradiation.^83,84^

**Figure 11:**
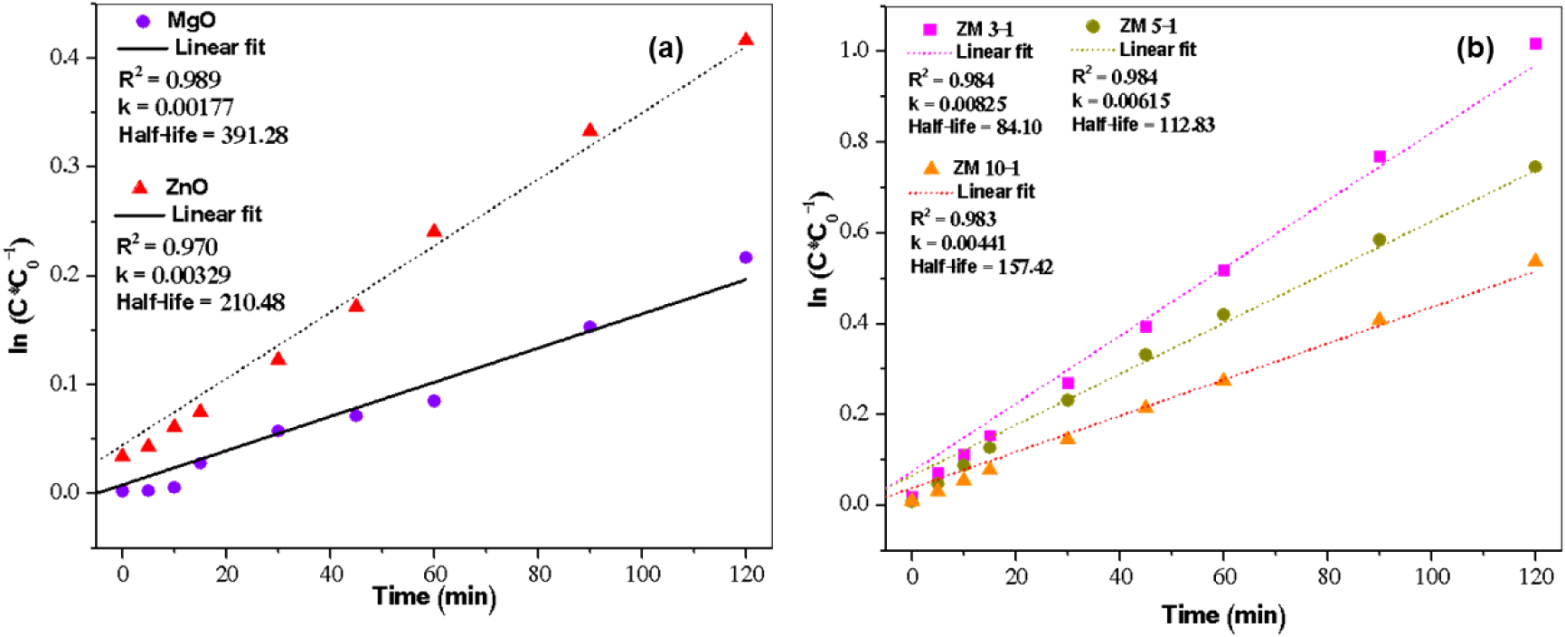
Kinetics of ZnO/MgO nanohybrid reactions: (a) pure nano-ZnO and MgO, (b) ZM 3–1, ZM 5–1, and ZM 10–1.

This behavior is attributed to the low initial pollutant concentrations and the abundance of active sites on the catalyst surface, which are typical conditions of heterogeneous photocatalysis. The improved performance and rate constants observed in these heterostructured systems are attributed to enhanced charge separation and increased generation of reactive species; however, the overall reaction order remains pseudo-first order in most cases.^85^ The *k* values for the photocatalysts were: ZnO (0.00329 min^–1^), MgO (0.00177 min^–1^), ZM 3–1 (0.00825 min^–1^), ZM 5–1 (0.00615 min^–1^), and ZM 10–1 (0.00441 min^–1^). The half-lives of the photocatalysts were ZnO (210.48 min), MgO (391.28 min), ZM 3–1 (84.10 min), ZM 5–1 (112.83 min), and ZM 10–1 (157.42 min). The half-life time of ZM 3–1 was shorter than that of the other photocatalysts, indicating a degradation rate in a much shorter time.

In Figure 12, the percentage of degradation, i.e., the pollutant removal efficiency, varied between 20% and 64%, exhibiting a synergistic trend between the increase in ZnO amount in the nanohybrids and the enhancement in caffeine degradation. ZM 3–1 was the sample that exhibited the most significant degradation capacity, degrading 64% of the caffeine. The peculiar photocatalytic activity of ZnO/MgO stems from its ability to capture ultraviolet radiation and produce potent oxidants, such as hydroxyl radicals (•OH), which can efficiently decompose contaminants in aqueous media.^86^ From these results, the combination of nano-ZnO and MgO facilitates better charge separation and reduces the recombination of electron–hole pairs, which is crucial for efficient photocatalysis.^87^ Studies suggest that rod-shaped ZnO nanoparticles have about 30% larger surface area and higher contaminant degradation efficiency than spherical ZnO nanoparticles, likely due to their morphology providing more active sites and better charge separation.^72^ The rod-shaped morphology of ZnO/MgO nanostructures demonstrates a significant impact on photocatalytic efficiency, as this elongated configuration provides a dual benefit: it increases the active surface area, increasing the contact interface with caffeine molecules and creating interparticle pores that facilitate adsorption,^88^ while simultaneously suppressing electron–hole recombination by enhancing charge separation and transfer.^59^ This synergy between maximizing reactive sites and optimizing carrier dynamics enhances the generation of oxidizing species, thereby increasing the degradation of the contaminant under UV irradiation.

**Figure 12:**
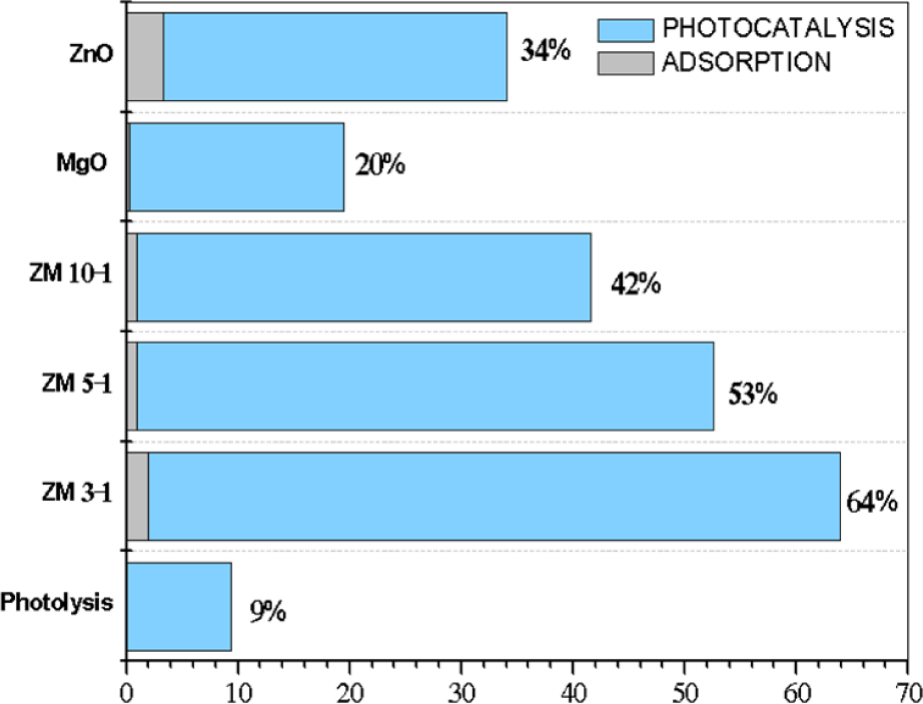
Percentage of caffeine degradation for nano-ZnO, nano-MgO, and the ZnO/MgO nanohybrids. Photolysis indicates the pollutant in the absence of the ZnO/MgO photocatalysts.

When comparing these results with the band gap energy results, the band gap values for ZM 5–1 and ZM 10–1 were close to pure ZnO, but their photocatalytic activities were much higher than ZnO, evidencing the influence of morphology on photocatalytic efficiency, since pure ZnO has a spherical morphology and ZM 5–1 and ZM 10–1 have a much greater predominance of rods. In ZM 3–1, a synergy exists between the decrease in band gap value and morphology/structure/defects, resulting in a significantly higher photocatalytic efficiency compared to other photocatalysts. The characterization results indicate that the superior performance of ZM 3–1 in contaminant elimination may be associated with its structure, morphology, and optical features, which could provide an enlarged surface area and defects induced by interfacial stress and dislocation densities.^56^ This expansion provides greater availability of reactive sites for dye adsorption and subsequent photochemical processes at the solid–liquid interface.^89^ In parallel, there may be optimization of charge carrier (electron/hole) transport, which enhances the generation of ROS through advanced oxidation,^90,91^ as ROS and Zn^2+^ ions drive higher photocatalytic activities.^92^ These results corroborate the calculations of the half-lives of the photocatalysts, indicating that the Zn-rich photocatalysts have a shorter half-life for degrading caffeine. It is observed that the sample that obtained the best performance, ZM 3–1, was also the one that had the shortest half-life.

The use of noble metal NPs to dope ZnO in heterogeneous photocatalysis improves the efficiency in the degradation of pollutants, such as caffeine. Vaiano et al.^93^ demonstrated excellent catalytic efficiency in removing caffeine from aqueous solutions using ZnO nanoparticles (NPs) modified with noble metals (Pt, Ag, and Au), achieving nearly complete degradation after 4 h. Recent studies of ZnO with Ag NPs showed efficiency around 80% in 160 min.^94^ The main objective of our work is precisely to avoid using metals to increase the “green chemistry” aspect of our synthesis. Since the materials are simple to synthesize, with inexpensive precursors, low energy consumption, short reaction time, and no metals needed, the fact that our work achieved approximately 64% efficiency in only 120 min is a great result.

Also, the addition of metals presents significant environmental challenges, including toxicity, persistence in the environment, and risks of bioaccumulation. Noble metal NPs, due to their high chemical stability, tend to persist in the environment after the photocatalytic process, especially in sediments or soils. In contrast, ZnO-based NPs can be cheaper, partially dissolve, and are less toxic to humans.^95,96^ The combination of ZnO with noble metals can alter toxicity. For example, doping with Ag can increase the release of metal ions, potentially increasing the impact on aquatic ecosystems. Replacing noble metals with less toxic dopants, such as nitrogen, carbon, and transition metals, or with hybrid nanostructures of materials, such as MgO, can reduce environmental impacts and costs.

## 4 Conclusions

This study successfully established a significant advancement in materials science through the novel, environmentally friendly synthesis of layered ZnO/MgO nanohybrids. Utilizing a one-pot, low-cost, low-temperature route, under 400 °C, based on a glycerol-urea hydrogel system, we developed a reproducible method to create these nanohybrids. Notably, this strategy avoids the use of noble metals or toxic surfactants, aligning the material design with sustainable and green chemistry principles. Characterization confirmed the formation of a ZnO/MgO heterostructures with rod-shaped morphology. Due to growing concerns over caffeine contamination in water and its adverse environmental impacts, this pollutant was chosen as proof of materials merit. The ZM 3–1 composite exhibited remarkable performance, degrading 64% of caffeine in 120 min, an efficiency almost two times greater than pure ZnO, attributed to the synergy between its ZnO and MgO heterostructure dominated by nanorods, which can increase the contact area with the contaminant and optimize the generation of reactive oxygen species. Additionally, advanced electronic properties and the suppression of crystalline lattice defects that inhibit electron–hole recombination were validated by Williamson-Hall and Williamson–Smallman analysis. These characteristics led to accelerated kinetics (*k* = 0.00825 min^–1^; *t*_1/2_ = 84.1 min), similar to those of conventional noble metal-based photocatalysts without their associated toxicological risks.

The one-pot synthesis, utilizing accessible precursors (Zn/Mg nitrates, isopropanol, and urea), offers scalable advantages, including reproducibility, energy efficiency, and the elimination of toxic surfactants. By integrating high photocatalytic performance, morphological innovation, and principles of the circular economy, these ZnO/MgO nanohybrids present a promising option for wastewater treatment, effectively addressing the global challenge of water contamination by emerging pollutants.

## Supplementary Material



## Notes

Emission lamp of a 125 W commercial mercury vapor lamp without a bulb (Figure S1); adsorption equilibrium of the samples in the dark (Figure S2); the maximum absorption wavelength of caffeine (Figure S3); HRTEM/EDXA images of ZnO/MgO with higher resolution (Figure S4); and TEM image of ZM 20–1 (Figure S5) (PDF)
