# Supplementary material for: Layered ZnO/MgO Nanohybrids for Photocatalytic Caffeine Degradation
Source: ACS Omega. 2026 Feb 10;11(7):12359–70. doi: 10.1021/acsomega.5c11898 (PMC12947214; doi:10.1021/acsomega.5c11898)

Supporting Information

Layered ZnO/MgO nanohybrids for photocatalytic caffeine degradation

Lorena Portela Brazuna[a*], Benjamim Sipauba Gonçalves Rubim[b], Rebeca Bacani[c], Josy A. Osajima[b], Eduardo Rezende Triboni[a]

a Universidade Federal de São Paulo, Campus São José dos Campos (UNIFESP-SJC) – Unidade Talim. Rua Talim, nº 330, São José dos Campos, São Paulo, CEP 12.231-280 – Brazil.

b Laboratório Interdisciplinar de Materiais Avançados (LIMAV). Universidade Federal do Piauí, Campus Ministro Petrônio Portela, Teresina, PI, CEP 64049-550 – Brazil.

c Escola de Engenharia de Lorena da Universidade de São Paulo, Departamento de Engenharia Química (DEQUI). Estrada Municipal do Campinho, 100, Lorena, SP - CEP 12.602-810 – Brazil.

**Corresponding Author.*

E-mail: lobrazuna@gmail.com

Figure S1. Emission Lamp of a 125 W commercial mercury vapor lamp without bulb.

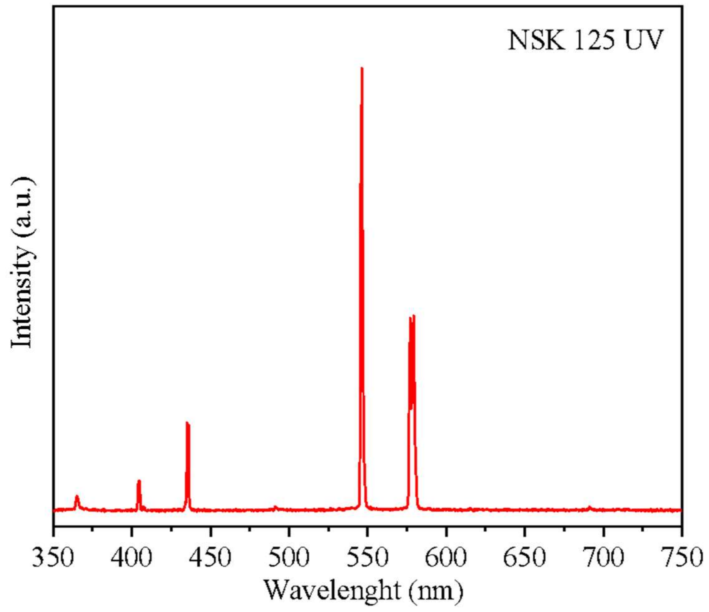

In our experiments, the photocatalytic tests were carried out using a 125 W commercial mercury vapor lamp without a bulb as a UV light source. The radiation intensity was 6.0 ± 0.2 μW cm^{-2}, measured using a radiometer (Hanna), with a main emission peak in the range of 350–450 nm (Figure S1). This type of lamp has already been employed in recent studies from our research group.[38-41]

Figure S2. Adsorption equilibrium of the samples in the dark.

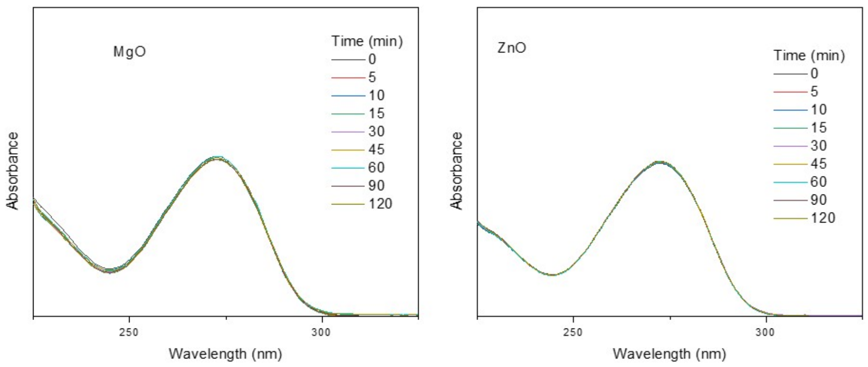

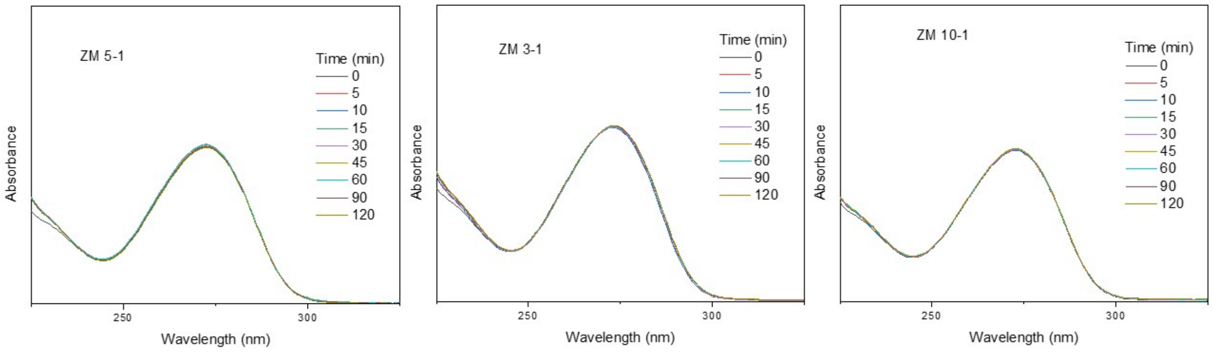

Before irradiation, the solutions were stirred in the dark for 30 minutes to establish the adsorption-desorption equilibrium between the catalyst and the contaminant. This procedure is commonly employed in photocatalytic studies to distinguish adsorption from photodegradation.

Figure S3. The maximum absorption wavelength of caffeine.

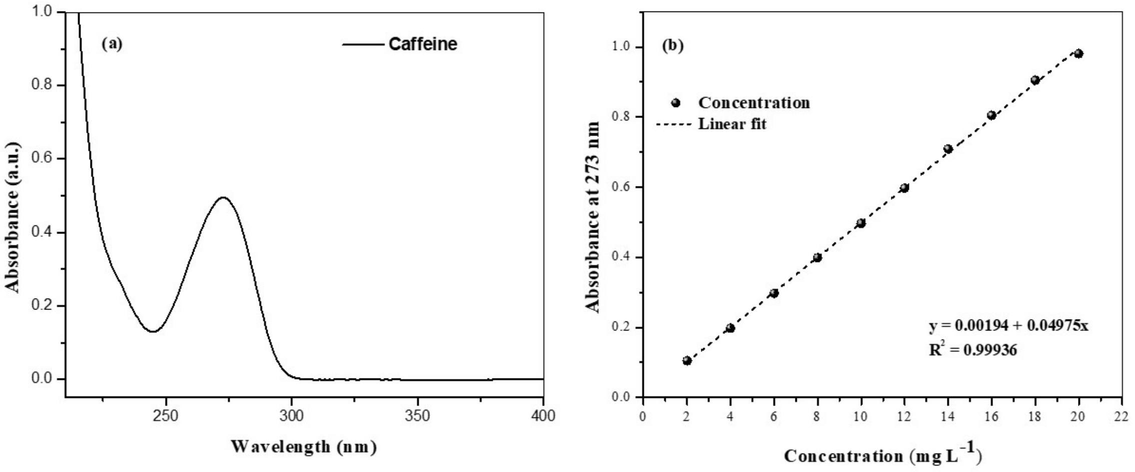

The maximum absorption wavelength of caffeine was determined by UV-Vis spectroscopy at 273 nm (Figure S2 (a)). A calibration curve was subsequently constructed at this wavelength (Figure S2 (b)).

Figure S4. HRTEM/EDXA images ZnO/MgO with higher resolution.

a) ZM 3-1: atomic composition via EDXA

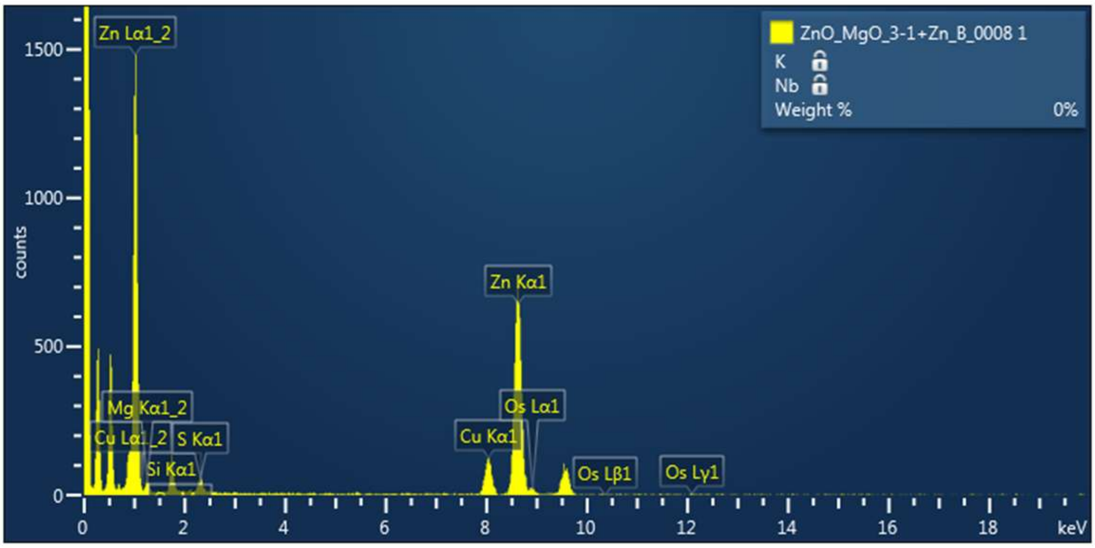

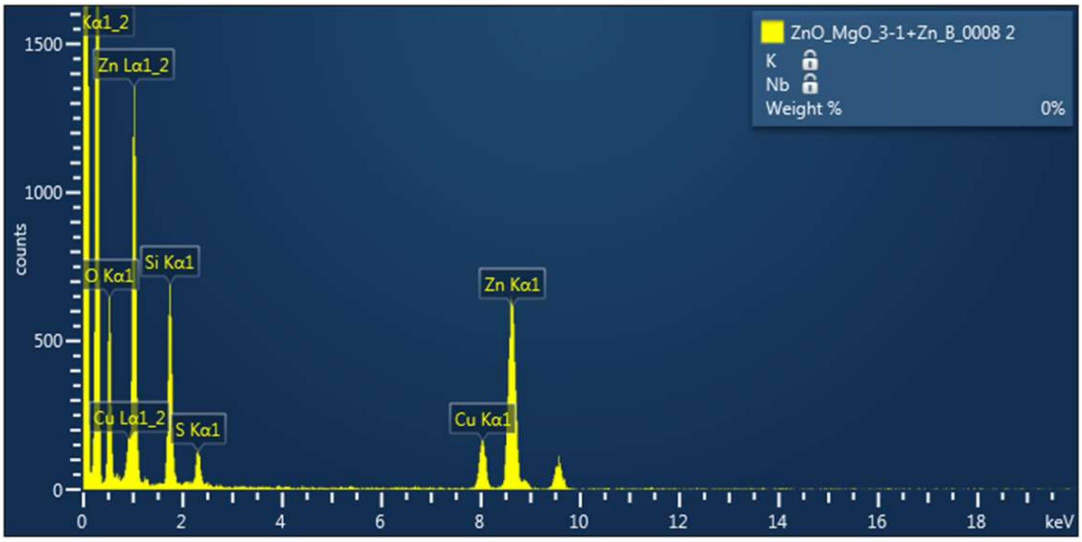

b) ZM 5-1: atomic composition via EDXA

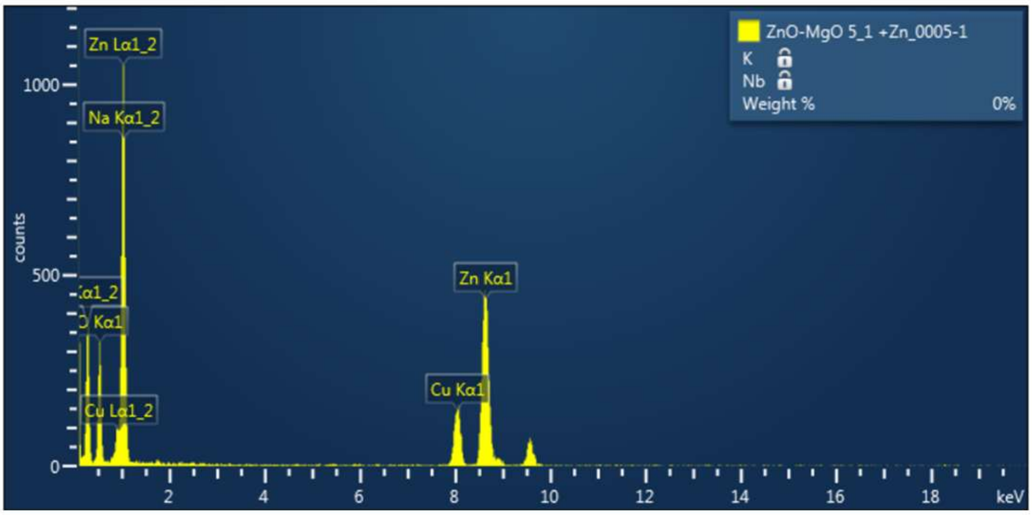

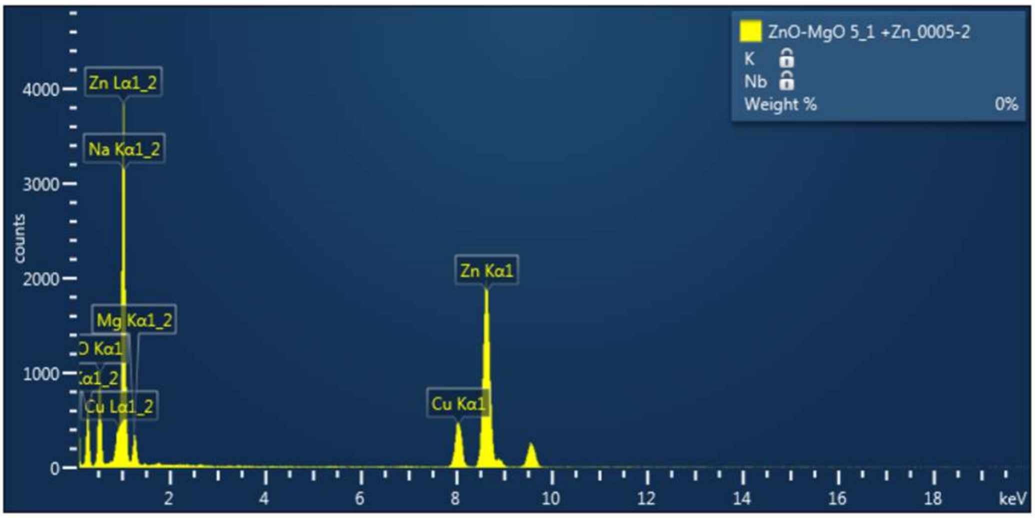

c) ZM 10-1: atomic composition via EDXA

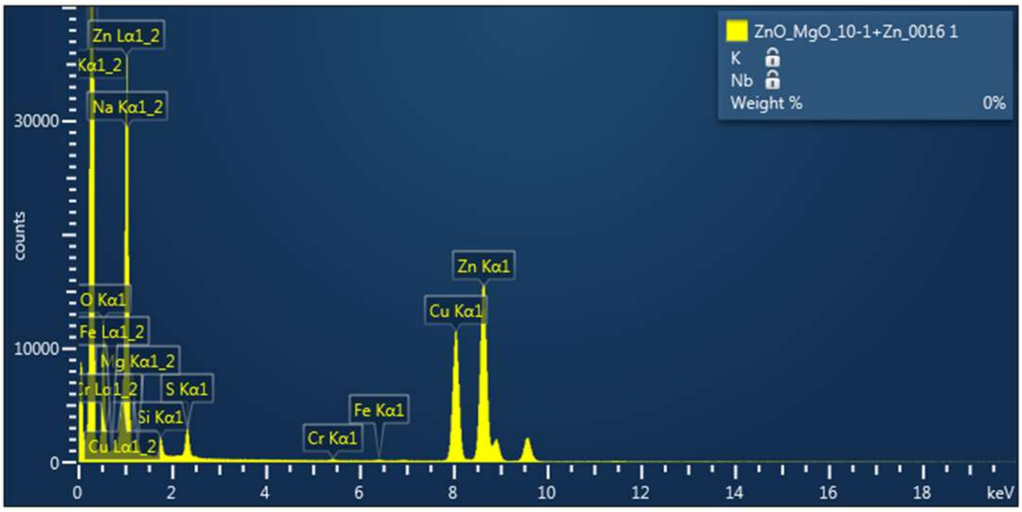

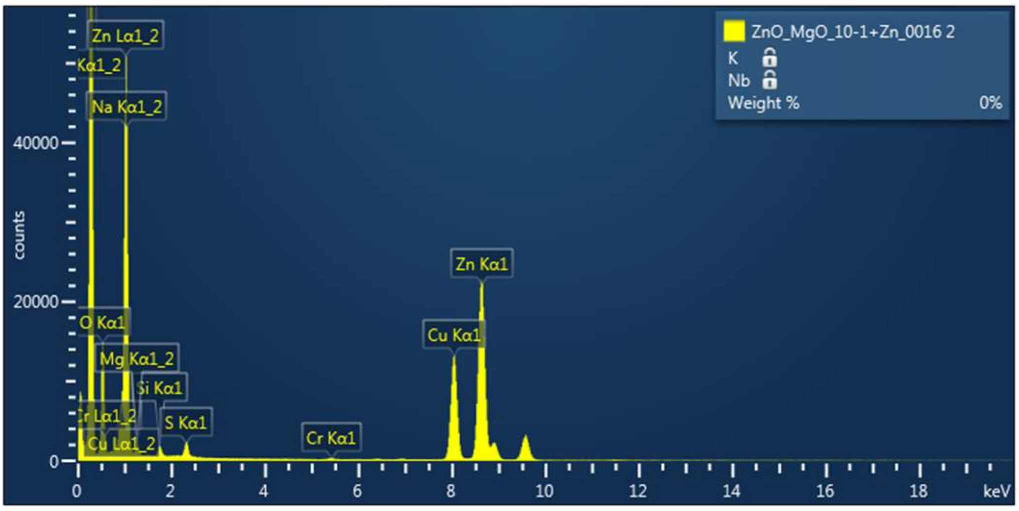

Figure S5. TEM image ZM 20-1.

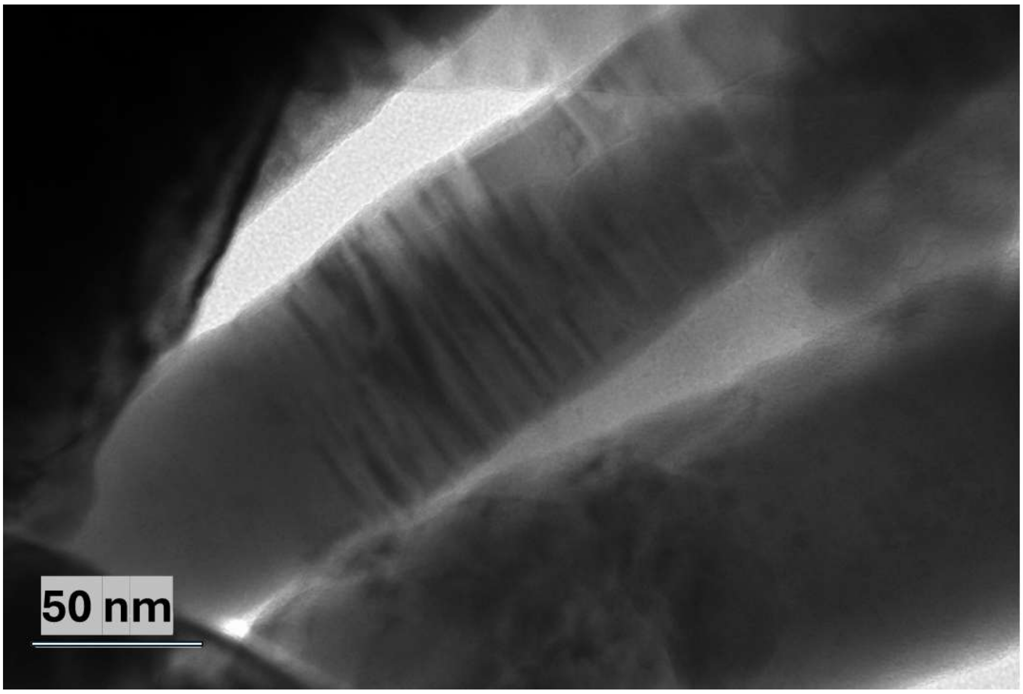

Supplement: Supplementary file 1 [file ao5c11898_si_001.pdf]
